# Advances and perspectives in the development of vaccines against highly pathogenic bunyaviruses

**DOI:** 10.3389/fcimb.2023.1174030

**Published:** 2023-05-18

**Authors:** Tong Chen, Zhe Ding, Jiaming Lan, Gary Wong

**Affiliations:** ^1^ Viral Hemorrhagic Fevers Research Unit, Chinese Academy of Sciences (CAS) Key Laboratory of Molecular Virology & Immunology, Institut Pasteur of Shanghai, Chinese Academy of Sciences (CAS), Shanghai, China; ^2^ University of Chinese Academy of Sciences, Beijing, China

**Keywords:** bunyaviruses, Crimean-Congo Hemorrhagic Fever Virus, Rift Valley Fever Virus, Hantaan virus, vaccines

## Abstract

Increased human activities around the globe and the rapid development of once rural regions have increased the probability of contact between humans and wild animals. A majority of bunyaviruses are of zoonotic origin, and outbreaks may result in the substantial loss of lives, economy contraction, and social instability. Many bunyaviruses require manipulation in the highest levels of biocontainment, such as Biosafety Level 4 (BSL-4) laboratories, and the scarcity of this resource has limited the development speed of vaccines for these pathogens. Meanwhile, new technologies have been created, and used to innovate vaccines, like the mRNA vaccine platform and bioinformatics-based antigen design. Here, we summarize current vaccine developments for three different bunyaviruses requiring work in the highest levels of biocontainment: Crimean-Congo Hemorrhagic Fever Virus (CCHFV), Rift Valley Fever Virus (RVFV), and Hantaan virus (HTNV), and provide perspectives and potential future directions that can be further explored to advance specific vaccines for humans and livestock.

## 1 Introduction

In 2019, the International Committee on Taxonomy of Viruses (ICTV) updated the classification list of Bunyaviruses from the family *Bunyaviridae* to the order *Bunyavirales*, to reflect the expanded diversity of these RNA viruses (Abudurexiti et al., 2019). According to the ICTV website (https://ictv.global/taxonomy), the order *Bunyavirales* includes 14 families, 4 subfamilies, 60 genera and 496 species as of September 2022. The bunyavirus genome consists of linear, segmented, negative-sense single stranded or ambisense RNA, except for non-enveloped plant tenuiviruses (Ter Horst et al., 2019). Generally, bunyavirus genomes are composed of the S segment encoding the nucleocapsid protein (NP), the M segment encoding the glycoprotein (GP), and the L segment encoding the RNA dependent RNA polymerase (RdRp). Some bunyaviruses are known to be highly virulent to humans and require manipulation in a high biocontainment laboratory, such as Crimean-Congo hemorrhagic fever virus (CCHFV) (Tsergouli et al., 2020; Temur et al., 2021), Rift Valley fever virus (RVFV) (Dar et al., 2013; McMillen and Hartman, 2021), and Hantaan virus (HTNV) (HTNV can be operated in a BSL-3 laboratory depending on viral concentration and animal species) (Knudsen et al., 1994; Dong et al., 2019; Meechan and Pots, 2020). These viruses belong to the families *Nairoviridae*, *Phenuiviridae*, and *Hantaviridae*, respectively. Due to globalization leading to increased travel and trade, human health is closely connected to animal and environmental health (Gruetzmacher et al., 2021). This can be partially linked to the changing environment and climate leading to the contraction of natural territories and the forced migration of wild animals and insects to human territories, resulting in increased chances for interaction of these wild species with humans and an elevated pathogen spillover risk.

Another factor to consider is outbreak preparedness for neglected pathogens that may pose substantial threats to humans due to their virulence and high mortality rates. Emerging viruses causing disease outbreaks that have garnered high-profile international attention since the 21^st^ century include but are not limited to: SARS-CoV, H1N1 “swine” influenza, MERS-CoV, Ebola virus, Zika virus, and SARS-CoV-2 (Shi et al., 2017; Yang et al., 2022). These viruses were either neglected or unknown at the beginning of the outbreak, and there is a possibility that other poorly characterized pathogens with high lethality rates could potentially cause widespread pandemics in the future. Bunyaviruses, such as CCHFV, RVFV and HTNV are understudied hemorrhagic fever viruses with potentially devastating public health consequences and there are concerns that these viruses could be suitable agents for bioweapons development (Flick and Whitehouse, 2005; Rolin et al., 2013; Tian and Stenseth, 2019). In addition to surveillance efforts, medical countermeasures, such as vaccines, monoclonal antibodies and other small-molecule therapeutics are also needed as preventative and treatment options.

As a method of proactive protection against viral infection, an appropriate vaccine is usually the primary choice. In the development of vaccines, however, there are important considerations, including its safety, immunogenicity, and efficacy in animals and humans, its ease and costs of production, and whether transmission from vaccinated hosts to others is possible. Other logistical considerations include the scale of vaccination (such as endemic and other at-risk populations) and access to the highest biocontainment, such as BSL-4 laboratories, to test these vaccine candidates. Besides traditional vaccines, such as inactivated and subunit vaccines, new emerging technologies have facilitated the innovation of novel vaccines, such as microfluidic-based mRNA vaccines (Tarim et al., 2023), and silico-based antigen design (Hederman and Ackerman, 2023; Kuri and Goswami, 2023), which have enriched the variety of potential vaccine candidates. Here, we summarize research advances on prophylactic options currently in development for three highly pathogenic bunyaviruses: CCHFV, RVFV and HTNV, and discuss future directions towards the clinical development of these vaccine candidates.

## 2 Vaccines against CCHFV

CCHFV is a genetically diverse tick-borne pathogen with seven clades (Asia-1, Asia-2, Africa-1, Africa-2, Africa-3, Europe-1 and Europe-2), and can be found in Asia, Africa and Europe, south of 50 degrees northern latitude (Fillâtre et al., 2019). Carried by *Hyalomma* ticks, CCHFV could directly infect humans *via* tick bite, or indirectly infect humans *via* contact with the blood or tissue of viremia-phase livestock, which amplify the virus after infection from a CCHFV-positive tick (Hoogstraal, 1979; Bente et al., 2013; Spengler et al., 2016; Papa et al., 2017). Case fatality rates vary based on different regions and outbreaks. For instance, 15 deaths from 480 cases (3%) in Turkey during 2020, and 19 deaths from 37 cases (51%) in India during 2019 (Kuehnert et al., 2021), but the general case fatality rate is usually considered to be around 30% (Sanchez et al., 2002; Papa et al., 2017; Tipih et al., 2021; Hawman and Feldmann, 2023). The envelope glycoproteins Gn and Gc of CCHFV are known to be the main immunogen and possess neutralizing epitopes, but some of these neutralizing epitopes not always conserved due to the genetic diversity of CCHFV (Ahmed et al., 2005; Wampande et al., 2021). Meanwhile, as a more conserved domain, NP could also be used as a target antigen against CCHFV (Karaaslan et al., 2021). Non-structural proteins, such as the secreted GP-38 glycoprotein, was verified through protective antibody studies to be another potential target for CCHFV vaccine candidates (Karaaslan et al., 2021).

### 2.1 Inactivated vaccines

In 1974, Bulgaria approved a whole-virion CCHFV inactivated vaccine, prepared from viruses grown in newborn mouse brain tissue, and then inactivated by chloroform, heated at 58°C and adsorbed onto aluminum hydroxide (Mousavi-Jazi et al., 2012). At-risk populations such as butchers and animal slaughter workers in Bulgaria are immunized with this candidate, but the efficacy of the Bulgarian vaccine is yet to be demonstrated in clinical trials (Mousavi-Jazi et al., 2012). Subsequently, advances in technology showed that certain cell lines, such as SW-13, VeroE6 and Huh7 were capable of replicating CCHFV to different levels, which provided possibilities to produce CCHFV-inactivated vaccines *via* alternate means (Dai et al., 2021).

Berber et al. (2021) showed that both inactivated vaccine produced by VeroE6 culture (CCVax) and sucking mice brains (MBVax) induced humoral responses and dose-dependent protection, but better performance was observed with CCVax (**Table 1**). Although CCHFV-specific antibody levels could be sustained for at least ten months after the final administration, Mousavi-Jazi et al. (2012) verified that high levels of CCHFV-specific antibodies did not necessarily correlate with neutralization activity (**Table 1**), a result which was further demonstrated in a study comparing the virus neutralization and CCHFV-specific IgG titers from both VeroE6- and suckling mouse brain-produced inactivated vaccines (**Table 1**) (Pavel et al., 2020). Additionally, mouse brains and cell lines, and embryonated chicken eggs (ECE) could also be used for CCHFV cultivation (Xia et al., 2013), but so far ECE has not been used for producing inactivated vaccines, possibly because inactivated RVFV produced from chick embryo induced a lower neutralization index (1.2 logs) compared to VeroE6 cells (2.3 logs) and mouse brains (2.8 logs) (Randall et al., 1962). The above vaccines use formalin for inactivation, but a study showed that inactivation by β-propiolactone (BPL) may retain higher immunogenicity (Lim et al., 2018), potentially demonstrating a method for the development of more effective inactivated CCHFV vaccines.

**Table 1:** Summary of CCHFV vaccine development in animals or volunteers.

Vaccine type	Antigen origin	Dose and regimens	Specific antibody	Neutralizing antibody	T-cell response	Challenge	Survival rate	Reference
Inactivated	Turkey-Kelkit06	5μg/10μg/20μg with alum adjubant per group 3 doses at 3 weeks intervals in BALB/C mice *via* IP route	Yes	Yes	NT	NT	NT	(Berber et al., 2021)
	Bulgarian	1 dose 30 to 55 years volunteers 4 doses 30 to 55 years volunteers	Yes	Yes	Yes	NT	NT	(Mousavi-Jazi et al., 2012)
	Turkey-Kelkit06	5μg/10μg/20μg with alum adjuvant per group 3 doses at 0, 14, 27 days in BALB/c mice *via* IP route	Yes	Yes	Yes	100 PFU Turkey-Kelkit06 at day 41	20μg group BALB/c 100%	(Pavel et al., 2020)
Subunit	Gn-e and Gc-e from IbAr10200	1.4μg Gc-e with Sigma Adjuvant System per group 2 doses at day 0 and 21 in STAT129 mice *via* IP route 7.5μg Gc-△e with Sigma Adjuvant System per group 2 doses at day 0 and 21 in STAT129 mice *via* IP route 15μg Gn-e 2 doses with Sigma Adjuvant System per group 2 doses at day 0 and 21 in STAT129 mice *via* IP route	NT	Yes	NT	100 PFU IbAr10200 at day 35	Gc-e group STAT129 mice 0%	(Kortekaas et al., 2015)
	eG_N_ and eG_C_ from IbAr10200	1μg/5μg/20μg A-G-eG_N_ with 201VG and Poly(I/C) adjuvant per group 4 doses at week 0, 3, 6, 9 in BALB/c mice *via* subcutaneous route 1μg/5μg/20μg A-G-eG_C_ with 201VG and Poly(I/C) adjuvant per group 4 doses at week 0, 3, 6, 9 in BALB/c mice *via* subcutaneous rout	Yes	ND Yes Yes	Yes	NT	NT	(Wang et al., 2022)
	Gn from GB: DQ446216.1	50 μl Sf9 cell lysate containing Gn with Freund’s adjuvant 3 doses at week 0, 2,4, in BALB/c mice	Yes	NT	Yes	NT	NT	(Rahpeyma et al., 2017)
	Gc and Gn from GB: DQ446215	Feeding 10μg Gc/Gn expressed by leaves 4 doses at 1 week interval in BALB/c Feeding 10μg Gc/Gn expressed by root 4 doses at 1 week interval in BALB/c	Yes	NT	NT	NT	NT	(Ghiasi et al., 2011)
VLPs	Gn, Gc and Np from IbAr10200	10^6^ VLPs 3 doses at week 0, 4, 7 in IFNAR^-/-^ mice *via* IP route	Yes	Yes	Yes	400 FFU IbAr10200 at week 13	A129 IFNAR^-/-^ mice 40%	(Hinkula et al., 2017)
VRPs	Gn and Gc from Hoti	10^6^ particles 3 doses at week 0, 2, 5, in BALB/cN mice *via* subcutaneous rout	Yes	Yes	NT	NT	NT	(Tran et al., 2022)
Viral vector	rVSV-Gpc	10^7^ PFU 1 dose at day 0 in STAT-1^-/-^ mice *via* IP route 10^7^ PFU 2 doses at day 0,14 in STAT-1^-/-^ mice *via* IP route	Yes	Yes	NT	50 PFU Turkey2004 at day 35	STAT-1^-/-^ mice 100%	(Rodriguez et al., 2019)
	Ad-5-Np from IbAr 10200	1.25×10^7^ IFU 1 dose at day 0 in IFNAR^-/-^ mice *via* IM route First dose 1.25×10^7^ IFU at day 0, Second dose 1.25×10^8^ IFU at day 28 in IFNAR^-/-^ mice *via* IM route	Yes	NT	NT	50 TCID_50_ IbAr 10200 at day 56	IFNAR^-/-^ mice 33% IFNAR^-/-^ mice 78%	(Zivcec et al., 2018)
	Ad-5-Np from Ank-2	100 TCID_50_ 2 doses at day 0, 14 in BALB/c mice *via* IP route	Yes	ND	Yes	1000 TCID_50_ Ank-2 at day 28	IFN^α/β/γR-/-^ mice 100%	(Aligholipour Farzani et al., 2019b)
	BoHV-△TK-Np from Ank-2	100 TCID_50_ 2 doses at day 0, 14 in BALB/c mice *via* IP route	Yes	ND	Yes	1000 TCID_50_ Ank-2 at day 28	IFN^α/β/γR-/-^ mice 100%	(Aligholipour Farzani et al., 2019b)
	MVA-Gp from IbAr 10200	10^7^ PFU 2 doses at day 0, 14 in A129 (IFN-α/βR^-/-^) mice *via* IM route	Yes	NT	Yes	200 TCID_50_ IbAr 10200 at day 28	A129 (IFN-α/βR^-/-^) mice 100%	(Buttigieg et al., 2014)
	MVA-Gp from IbAr 10200	10^7^ PFU 2 doses at day 0, 14 in A129 (IFN-α/βR^-/-^) mice *via* IM route	Yes	NT	Yes	200 FFU IbAr 10200 at day 28	A129 (IFN-α/βR^-/-^) mice 100%	(Dowall et al., 2016b)
	MVA-Np from IbAr 10200	10^7^ PFU 2 doses at day 0, 14 in A129 (IFN-α/βR^-/-^) mice *via* IM route	Yes	NT	Yes	200 TCID_50_ IbAr 10200 at day 28	A129 (IFN-α/βR^-/-^) mice 0%	(Dowall et al., 2016a)
DNA	Np from Ank-2	50μg pV-N13 2 doses at day 0, 14 in BALB/c mice *via* IM route 40μg pV-N13 + 10μg pCD24 at day 0, 14 in BALB/c mice *via* IM route	Yes	ND	Yes	1000 TCID_50_ Ank-2 at day 28	IFNAR ^-/-^ mice 100%	(Aligholipour Farzani et al., 2019c)
	Np from Ank-2	50μg pCD-N1 2 doses at day 0, 14 in BALB/c mice *via* IM route	Yes	ND	Yes	1000 TCID_50_ Ank-2 at day 28	IFN^α/β/γR-/-^ mice 75%	(Aligholipour Farzani et al., 2019b)
	M segment from IbAr 10200	25μg CCHFV-M 3 doses at 3 weeks intervals in IFNAR ^-/-^ mice *via* IM route 25μg CCHFV-M 3 doses at 3 weeks intervals in C57BL/6 mice *via* IM route	Yes	Yes	NT	100 PFU IbAr 10200 at week 10	IFNAR ^-/-^ mice 71% IS C57BL/6 mice 60%	(Garrison et al., 2017)
	S and M segment from Hoti	1mg Np-ubiquitin+1mg GPc-ubiquitin 3 doses at 3 weeks intevals in Cynomolgus macaque *via* IM route	Yes	Yes	Yes	10^5^ TCID_50_ Hoti at day 63	Cynomolgus macaque NT	(Hawman et al., 2021a)
	Np and GPc from Hoti	1mg Np+1mg GPc 2 doses at day 0,21 in macaque *via* IM route 1mg Np 3 doses at day 0, 21, 42 in macaque vai IM route 1mg GPc 3 doses at day 0, 21, 42 in macaque vai IM route	Yes Yes ND	Yes ND Yes	Yes ND Yes	10^5^ TCID_50_ Hoti at day 42 10^5^ TCID_50_ Hoti at day 63 10^5^ TCID_50_ Hoti at day 63	Macaque NT	(Hawman et al., 2022)
mRNA	Np from Ank-2	25μg mRNA-Np 1doses at day 0 in C57BL/6 vai IM route 25μg mRNA-Np 2 doses at day 0,14, in C57BL/6 vai IM route	Yes	ND	Yes	1000 TCID_50_ Ank-2 strain at day 42	IFNα/β/γR^-/-^ mice 50% IFNα/β/γR^-/-^ mice 100%	(Aligholipour Farzani et al., 2019a)
	Np and Gp from IbAr 10200	10μg mRNA-Np 2 doses at day0, 21 in IFNAR ^-/-^ mice *via* IM route 10μg mRNA-Gp 2 doses at day0, 21 in IFNAR ^-/-^ mice *via* IM route 10μg mRNA-Np+10μg mRNA-Gp 2 doses at day0, 21 in IFNAR ^-/-^ mice *via* IM route	Yes	ND Yes Yes	Yes	400 FFU IbAr 10200 at day 56	IFNAR ^-/-^ mice 100%	(Appelberg et al., 2021)
	Np and Gp from Hoti	2.5μg repNP 2 doses at day 0,28 in C57BL6/J mice *via* IM route 2.5μg repGPC 2 doses at day 0,28 in C57BL6/J mice *via* IM route 2.5μg repNP +repGPC 2 doses at day 0,28 in C57BL6/J mice *via* IM route	Yes	ND Yes Yes	Yes	100 TCID_50_ UG3010 at day 56	C57BL6/J treated with MAR1-5A3 mice 100% C57BL6/J treated with MAR1-5A3 mice 37.5% C57BL6/J treated with MAR1-5A3 mice 100%	(Leventhal et al., 2022)

ND indicates not detected, NT indicates not tested, GB indicates GenBank accession number, PFU indicates plaque forming unit, FFU indicates focus-forming units, IS indicates immuneosuppressed, IFU indicates infectious units, IP indicates intraperitoneal, IM indicate intramuscular.

### 2.2 Subunit vaccines

Subunit vaccines are based on the expression of recombinant proteins, in which segments of the viral antigen are generated, and contains advantages such as the possibility for high purity, minimal side effects, ease of large-scale production and low manufacturing costs. Adjuvants are used in conjunction to overcome shortcomings in this approach, such as low immunogenicity.

With the development of big data and artificial intelligence, bioinformatics is playing an increased role in the screening and design of suitable subunit vaccine targets. Using these *in silico* approaches, Sana et al. (2022) designed a subunit vaccine targeting all the seven genotypes of CCHFV, Nosrati et al. (2019) selected B/T cell epitopes based on parameters for antigenicity, allergenicity, toxicity, water solubility, hydrophobicity, population coverage, 3D structure and adjuvant compatibility, and Khan et al. (2021) constructed a 468-amino acid subunit vaccine, covering 98% of known CCHFV isolates. However, these subunit vaccine candidates have not yet been functionally evaluated in the laboratory, and as such the immunogenicity and efficacy is not known.

Traditional subunit vaccine approaches against CCHFV typically target the envelope glycoproteins Gn and Gc, or the more conserved NP. Golden et al. (2019) showed that the glycoprotein GP38, a region of the CCHFV GP, is not only secreted, but also becomes localized to the viral envelope and cellular plasma membranes, suggesting another potential target for vaccine development. A study comparing the efficacy of extracellular Gc (eGc), extracellular Gn (eGn), and conserved neutralizing antibody region of Gc (G-nAb), using a gram-positive enhancer matrix-protein anchor (GEM-PA) surface display system, found that these candidates could elicit humoral and cellular immunity in BALB/c mice, but specific neutralizing antibodies (nAbs) were only detected in the eGc group (**Table 1**) (Wang et al., 2022). Interesting, the eGn subunit vaccine generated by the *Drosophila* insect cell line could successfully elicit neutralizing antibodies (nAbs), but the levels were lower than that of the Gc ectodomain lacking the stem region (**Table 1**) (Kortekaas et al., 2015). These results suggest that different protein expression systems may also result in different immune responses. Rahpeyma et al. (2017) used a baculovirus expression system to generate Gn, showing that it was highly immunogenic and could elicit both high titers of antigen-specific antibodies and T-cell responses (**Table 1**), but it was not known whether these antibodies were neutralizing. Ghiasi et al. (2011) investigated the oral immunogenicity of a plant-derived G1 (Gc)/G2 (Gn) glycoprotein of the CCHFV with the aim of producing a CCHFV vaccine for livestock. From BALB/c mice studies, they found that BALB/c mice fed and boosted with transgenic plants expressing the CCHFV envelopes elicited strong levels of specific IgG and IgA antibodies in their serum and feces (**Table 1**), demonstrating that edible vaccines may be feasible against CCHFV, but these results need to be validated for protective efficacy in real livestock.

### 2.3 VLPs/VRPs vaccines

While straightforward to produce, the generation of inactivated vaccines for highly virulent pathogens require live virus to be grown to high titers, in which accidental laboratory release could result in a major public health incident. Virus-like particles (VLPs), which do not incorporate the viral genome of these pathogens and thus cannot replicate, addresses this weakness. Theoretically, VLPs maintain the structural integrity of the native virion, thus retaining the immunogenicity observed from inactivated vaccines. Viral replicon particles (VRPs) are VLPs that also include the complete S, L viral genome segments, but lack the M segment entirely. Thus, VRPs could enter host cells, but cannot produce nascent progeny particles.

Few VLPs vaccines against CCHFV have been reported thus far. In 2011, Zhou et al. (2011) successfully developed CCHFV VLPs *via* the expression of NP in insect cells using a recombinant baculovirus system, but did not test its immune response in animal models. In 2017, Zivcec et al. (2017) successfully used VLPs to screen nAbs against CCHFV, which supported that VLPs to have potential to induce efficient nAbs as a vaccine. In the same year, transcriptionally competent virus-like particles (tc-VLPs) elicited strong CCHFV-specific antibodies and nAbs, and found that the T cell response was biased towards Th2 after cytokine response analysis (Hinkula et al., 2017). However, the observation of significant weight loss, high RNA loads in the blood, spleen, and liver, as well as a protection rate of 40% in immunized mice (**Table 1**) (Hinkula et al., 2017), suggests that Th1-medidated responses may also play an important role in survival against CCHFV infection. Tran et al. (2022) developed a recombinant Kunjin strain of West Nile Virus (WNV_KUN_) replicon expressing CCHFV Gn and Gc, but found that this vaccine only induced seroconversion to WNV proteins and limited seroconversion of CCHFV-specific antibodies, without strongly nAbs against CCHFV (**Table 1**). Scholte et al. (2019) investigated a CCHFV VRPs vaccine, and showed that IFNAR^-/-^ mice inoculated with this candidate at 32 days before challenge was fully protective (**Table 1**), and a follow-up study by Spengler et al. (2021) showed that mice inoculated with the VRPs vaccine was fully protective when administered 14 and 7 days before challenge, whereas mice given the vaccine at 3 days before challenge exhibited disease symptoms, but fully recovered with 100% survival.

### 2.4 Viral-vectored vaccines

Viral-vectored vaccines are recombinant viruses which expresses the target antigen *via* the insertion of the gene into the genome of the vaccine virus backbone. Since these are typically live vaccine viruses, this platform can induce strong antigen-specific T/B-cell responses and can be easily grown to high titers *in vitro*. Despite these advantages, drawbacks include a lowered safety profile, especially for immunocompromised patients, and the risk of viral integration into the human genome, except for platforms comprising of non-segmented, negative-strand RNA viral vectors or virus that replicate in the cytoplasm.

Vesicular stomatitis virus (VSV) is a popular viral vector for the development of vaccines against emerging pathogens. Several VSV-based vaccines have been developed, including against influenza virus (Furuyama et al., 2020), SARS-CoV-2 (Malherbe et al., 2021), and Ebola virus, which received clinical approval from the World Health Organization (WHO) in late 2019 (Suder et al., 2018). The feasibility of VSV particles expressing CCHFV glycoproteins was demonstrated by Shtanko et al. (2014), supporting the possibility of characterizing this vaccine. Suda et al. (2016) then showed that VSV expressing Gn and Gc have some N-glycosylated sites and are likely to bind to C-type lectins like DC-SIGN, suggesting that these recombinant VSV can enter cells. Rodriguez et al. (2019) verified that VSV expressing CCHFV GP can indeed induce strong levels of specific antibodies and nAbs, as well as protect mice after challenge (**Table 1**).

In the development of viral vector vaccines, Adenovirus (Ad) is a popular platform that has been tested in clinical trials for a wide variety of infectious and genetic diseases. A human Ad serotype 5 (Ad5) expressing CCHFV NP was developed and found to stimulate NP-specific antibodies and elicit 78% protection after 2 doses (**Table 1**) (Zivcec et al., 2018). In contrast, Farzani et al (Aligholipour Farzani et al., 2019b). found that the Ad5-based vaccine expressing NP vaccine could induce strong T-cell and humoral responses with no nAbs detected, but 100% of mice were protected after a CCHFV challenge (**Table 1**). After the comparison of above two Ad5-NP vaccines, it was suspected that the different development processes of the vaccines may have led to a difference in efficacy, because the Ad5-NP vaccine with 100% protection was generated by AdMax™HI-IQ Kit J, whereas the other study used Adeno-X Adenoviral System 3 (Zivcec et al., 2018; Aligholipour Farzani et al., 2019b). In any case, the feasibility of Ad-based vaccines against CCHFV need to be further explored.

Modified vaccinia virus Ankara (MVA) is another well-established vaccine platform which has been demonstrated to be safe in humans (von Krempelhuber et al., 2010; Frey et al., 2014; Greenberg et al., 2015). Buttigieg et al. (2014) first demonstrated that an MVA-based vaccine expressing CCHFV GP protects 100% of mice from CCHFV infection, but it is not certain whether this vaccine induces nAbs (**Table 1**). Dowall et al. (2016b) further verified that MVA-based vaccines stimulate both arms of the immune system, which were required to elicit protective effects against lethal CCHFV challenge (**Table 1**). An MVA-based vaccine expressing NP was shown to induce humoral and cellular responses, but in contrast to Ad5-NP, the vaccine failed to protect mice from challenge (**Table 1**) (Dowall et al., 2016a).

The Bovine Herpesvirus Type 4 (BoHV-4) virus-based vector vaccine has been successfully explored for different pathogens, such as Ebola virus (Rosamilia et al., 2016). Farzani et al (Aligholipour Farzani et al., 2019b). reported that BoHV4-ΔTK expressing CCHFV NP could provide complete protection against CCHFV in IFN ^α/β/γ R−/−^ mice, and in the T-cell and passive antibody transfer assay, both arms of the immune response were shown to be required for protection (Dowall et al., 2016b).

### 2.5 DNA vaccines

DNA vaccines are typically delivered into cells through electroporation, in which its advantages include its speed and ease of design and production, making this platform an ideal candidate for rapid development of vaccines during outbreaks, especially against pathogens with high mutation rates. However, concerns with DNA vaccines include the possibility of the DNA vaccine integrating into the host genome, and low immunogenicity (Schalk et al., 2006; Dowall et al., 2016b).

There are various studies on CCHFV DNA vaccines that have demonstrated promising results. A study with pV-N13 vaccination expressing NP with pCD24 adjuvant showed that it could induce strong T-cell responses and protect 100% of IFNAR^-/-^mice from CCHFV infection, despite the lack of nAbs (**Table 1**) (Aligholipour Farzani et al., 2019c). However, another study showed that an unadjuvanted pCD-N1 vaccine protected 75% of IFN^α/β/γ-/-^ mice from challenge (**Table 1**) (Aligholipour Farzani et al., 2019b), suggesting that adjuvants are needed for optimal protection with DNA-vectored vaccines. Garrison et al. (2017) showed that an unadjuvanted DNA vaccine expressing CCHFV GP stimulated a balanced Th1/Th2 response, but was not sufficient for complete protection against CCHFV challenge, with only a 60% survival in mice (**Table 1**). However, a mixed DNA vaccine expressing CCHFV GP and NP showed a stronger Th1-immunity compared to Th2-immunity, and conferred 100% survival in mice (Hinkula et al., 2017). The mixed DNA vaccine with NP and GP precursor also protected cynomolgus macaques from CCHFV challenge (**Table 1**) (Hawman et al., 2021a), suggesting a crucial role for Th1 immunity in protection from CCHFV with DNA-vectored vaccines. Surprisingly, it was shown that DNA plasmids expressing NP and GPC stimulated Th2- and Th1-immunity (**Table 1**) (Hawman et al., 2022), perhaps due to the ubiquitin sequence playing an adjuvant role. Regardless, a mixed DNA vaccine with two doses eliciting both T- and B-cell responses was shown to be superior to even three immunizations with either NP or GPC alone (Hawman et al., 2022).

### 2.6 mRNA vaccines

Since the emergence of SARS-CoV-2, mRNA-based vaccines have been widely used in the human population. The advantages of mRNA vaccines include its good safety record, its speed and ease of design against emerging pathogens with high mutation rates, and good immunogenicity. However, mRNA vaccines are less stable than other types of vaccines, although a thermostable mRNA vaccine has been reported (Zhang et al., 2020), no commercial vaccines have been produced by this method so far. At the moment, mRNA vaccine still requires cold-chain storage and delivery, potentially contributing to logistical difficulties in less-developed regions with poor medical resources.

mRNA-vectored vaccines have also been tested against CCHFV since 2019. Farzani et al (Aligholipour Farzani et al., 2019a). found that an mRNA vaccine expressing NP could induce strong levels of IFN-γ, IL-4 and specific antibody but no nAbs, resulting in 100% protection in mice (**Table 1**). Appelberg et al. (2021) then compared 3 mRNA vaccines expressing either GnGc, NP or GnGc+NP, demonstrating that all candidates elicited T- and B-cell responses and completely protected IFNAR^-/-^ mice from CCHFV challenge (**Table 1**). Leventhal et al. (2022) established an alphavirus-based replicon mRNA (repRNA) vaccine expressing either NP, GPC or NP+GPC, and showed that both NP and the NP+GPC combined vaccine could confer complete protection against CCHFV, but the survival in the GPC group was suboptimal at 40% in C57BL6/J mice treated with MAR1-5A3, which blocks type I IFN receptor to further block type I IFN signaling (**Table 1**).

## 3 Vaccines against RVFV

RVFV was first reported from Kenya during 1930 (Daubney and Garnham, 1931). Since then, the virus has been found from Eastern Africa to Southern Africa, West Africa, the Egyptian delta, and the Saudi Arabian peninsula (Weaver and Reisen, 2010), and could be imported by travel from these endemic regions, such as the discovery of a RVFV-infected patient in China during 2016 (Liu et al., 2016). The transmission of RVFV depends on mosquitoes, which are the reservoir hosts, and amplified by intermediate hosts including sheep, cattle, goats, and camels, subsequently infecting humans typically *via* direct contact (Linthicum et al., 2016). The case fatality rate in humans can range between 20–50% (Dar et al., 2013).

Similar to CCHFV, the surface glycoproteins Gn and Gc play an important role in virus attachment to initiate infection, and as the main immunogen, has the ability to elicit nAbs (Dessau and Modis, 2013; Wu et al., 2017; Wang et al., 2019). The RVFV NP (3.5% variable in the nucleotide sequence level) is known to be more conserved and could induce strong T-cell responses (Bird et al., 2007; Xu et al., 2013), but the L protein is the most conserved among 33 different RVFV strains (Bird et al., 2007). Currently circulating strains of RVFV are descended from an ancestral species discovered during the 19^th^ century (Ikegami, 2012).

### 3.1 Inactivated vaccines

Randall et al. (1962) first developed formalin-inactivated RVFV candidate vaccines and indicated that the vaccine produced from mouse brains, which successfully elicited specific-RVFV antibodies, is better than those from primary monkey kidney cells and chicken eggs in both adult and sucking mice. The inactivated Entebbe strain of RVFV has been used by volunteers (Ikegami, 2019) but 6% of volunteers were reported to have mild side-effects such as headache, tiredness, and a general feeling of discomfort (Niklasson, 1982). To improve on this candidate, the United States Army Medical Research Institute of Infectious Diseases developed an inactivated RVFV vaccine by FRhL-2 cells called TSI-GSD-200 (Faburay et al., 2017). From its Phase I clinical trial results, although TSI-GSD-200 has better immunogenicity, mild and transient local reactions were still present, ranging from 5% at the lowest dose level to 43% at the highest, indicating safety concerns if the vaccine was to be administered to a larger population (Kark et al., 1982). Interestingly, there are long-lived RVFV-specific T-cell responses detected in volunteers vaccinated with the formalin-inactivated RVFV vaccine, suggesting that T-cell immunity may play a role in protection from RVFV infection (Harmon et al., 2020). Ronchi et al. (2022) formulated an inactivated Namibian field strain of RVFV with Montanide Pet Gel A and found after two doses, all animals seroconverted (**Table 2**). Due to the weak immunogenicity elicited by inactivated vaccines, different adjuvants were tested to determine which combination will elicit the strongest immune responses. Compared to alum, chitosan nanoparticles were found to be superior for inducing better immune responses against RVFV (**Table 2**) (El-Sissi et al., 2020). The foot and mouth disease (FMD)/RVF combined inactivated vaccine with oil adjuvant also elicited high antibody titers and strong T-cell responses, and could constitute a cheaper and more effective way to prevent RVFV in livestock (**Table 2**) (Gamal et al., 2014).

**Table 2:** Summary of RVFV vaccine development in animals.

Vaccine type	Antigen origin	Dose	Specific antibody	Neutralizing antibody	T-cell response	Challenge	Efficacy survival	Reference
Inactivated	Namibia_2010 from Vero E6 cells	0.3mg iRVFV with Montanide PET Gel A 2 doses at day 0,28 in sheep *via* subcutaneously	Yes	NT	NT	NT	NT	(Ronchi et al., 2022)
	Pan Tropic-Menya from BHK-211	6.4μg RVFV-CNP 2 doses at 2 weeks interval in Swiss albino mice *via* dorsal skinfold 6.4μg RVFV-CS 2 doses at 2 weeks interval in Swiss albino mice *via* dorsal skinfold 6.4μg RVFV-Alum 2 doses at 2 weeks interval in Swiss albino mice *via* dorsal skinfold 6.4μg RVFV-AG 2 doses at 2 weeks interval in Swiss albino mice *via* dorsal skinfold route	Yes	Yes	Yes	NT	NT	(El-Sissi et al., 2020)
	ZH 501 from BHK-21	0.2ml vaccine group with Montanide ISA 206 adjuvant 2 doses at day 0,7 in Swiss Albino suckling mice *via* IP route	Yes	Yes	NT	NT	NT	(Gamal et al., 2014)
Attenuated	rMP-12-PTNSs rMP-12-SFSNSs	10^5^ PFU 1 doses in CD-1 outbred mice *via* subcutaneously 10^5^ PFU 1 dosesin CD-1 outbred mice *via* subcutaneously	Yes	Yes	NT	10^3^ PFU wtZH501 at day 45	CD-1 mice 78% CD-1 mice 89%	(Lihoradova et al., 2013)
	rMP-12-C13type rMP-12-mPKRN167	10^5^ PFU 1 doses at post-infection 20min in C57BL/6 mice *via* subcutaneously 10^5^ PFU 1 doses at post-infection 20min in C57BL/6 mice *via* subcutaneously	NT	NT	NT	10^3.3^ PFU ZH501 at day 0	C57BL/6 mice 70% C57BL/6 mice 80%	(Gowen et al., 2013)
	rMP-12-TOSNSs	10^5^ PFU 1 doses in CD-1 mice *via* subcutaneously	Yes	Yes	NT	10^3^ PFU wtZH501 at day 45	CD-1 mice 100%	(Indran et al., 2013)
	rMP-12-GM50	5×10^5^ PFU 1 doses in CD-1 mice *via* subcutaneously	NT	Yes	NT	10^3^ PFU rZH501 at day 45	CD-1 mice 100%	(Ly et al., 2017b)
	rZH501-△NSs rZH501-△NSs-△NSm	5 Log_10_ PFU 1 doses in marmosets *via* subcutaneously	Yes	Yes	NT	6 Log_10_ PFU ZH501 at day 35	Marmosets NT	(Smith et al., 2018)
	scMP-12	10^5^ PFU 1 doses in CD-1 *via* IM route	NT	Yes	NT	10^3^ PFU ZH501 at day 40	CD-1 mice 90%	(Murakami et al., 2014)
	scMP-12-mutNSs	10^5^ PFU 1 doses in CD-1 *via* IM route	Yes	Yes	NT	10^3.3^ PFU ZH501 at day 40	CD-1 mice 100%	(Terasaki et al., 2018)
Subunit	eGn and Gc from ZH548	50μg eGn+50μg Gc with montanide ISA25 water-in-oil adjuvant 2 doses at day 0,21 in sheep *via* subcutaneously	Yes	Yes	NT	NT	NT	(Faburay et al., 2014)
	eGn and Gc	50μg eGn+50μg Gc with ISA25 VG adjuvant 2 doses at day 0, 21 in sheep *via* subcutaneously	Yes	Yes	NT	10^6^ PFU of Ken06 at day 35	Sheep (Dorper x Katahdin cross) 100%	(Faburay et al., 2016)
	eGn and Gc	50μg eGn+50μg Gc with ISA25 VG adjuvant 2 doses at day 0,21 in cattle *via* subcutaneously 50μg eGn 2 doses with ISA25 VG adjuvant 2 doses at day 0,21 in cattle *via* subcutaneously	Yes	Yes	NT	10^6^ PFU of Ken06 at day 35	Cattle (Holstein Friesian breed) NT	(Wilson et al., 2021)
	eGn from M35/74	10μg eGn with Stimune adjuvant 2 doses at day 0,21 in BALB/c mice *via* IP route	Yes	Yes	NT	10^2.7^ TCID50 M35/74 at day 42	BALB/c mice 100%	(de Boer et al., 2010)
	Gn head	20μg eGn_head_ with Stimune adjuvant 2 doses at day 0, 14 in BALB/cAnNCrl mice *via* IM route	NT	Yes	NT	10^3^ TCID50 re35/74 at day 28	BALB/c mice 100%	(Wichgers Schreur et al., 2021b)
	Gn head from MP12	50μg RVFV-BLPs 3 doses at week 0, 2, 4 in BALB/c mice *via* IM route	Yes	Yes	Yes	NT	NT	(Zhang et al., 2022)
VLPs	Gn and Gc from M35/74	10μg VLP with Stimune adjuvant 2 doses at day 0, 21 in BALB/c mice *via* IP route	Yes	Yes	NT	10^2.7^ TCID_50_ M35/74 at day 42	BALB/c mice 100%	(de Boer et al., 2010)
	Gn, Gc and Np from ZH501	15μg VLPs with Freund’s adjuvant 2 doses at week 0, 2 in mice *via* IM route	Yes	Yes	Yes	NT	NT	(Li et al., 2020)
	Glycoprotein and Np from MP12	6μg VLPs with Sigama Adjuvant System 3 dose at 9 days interval in BALB/c mice *via* subcutaneously 1ml chimVLPs with Sigama Adjuvant System 3 dose at 2 weeks interval in Wistar-Furth rats *via* subcutaneously	NT	Yes	Yes	10^3^ PFU ZH501 10^5^ PFU ZH501 at day 74	BALB/c mice 68% Wistar–Furth rats 100%	(Mandell et al., 2010)
	Gne and Gc from M35/74	5μg primary dose at day 0, 10μg second dose at day 13 and 10ug third dose at day 27 Gne-HA in BALB/c mice *via* saphenous vein	Yes	Yes	NT	NT	NT	(Mbewana et al., 2019)
	Gn and Gc	10^5.8^ TCID_50_ NSR-Gn 1 dose in BALB/c mice *via* IM route 10^5.8^ TCID_50_ NSR and 10^2.8^ TCID_50_ NDV 1 dose in BALB/c mice *via* IM route 10^2.8^ TCID_50_ NDV-GnGc-△F 1 dose in BALB/c mice *via* IM route	NT	Yes	NT	10^2.8^ TCID_50_ 35/74 at week 3	BALB/cAnCrl mice 100% BALB/cAnCrl mice 100% BALB/cAnCrl mice 0%	(Wichgers Schreur et al., 2014a)
Viral vector	Gn and Gc from Mp12	10^7^ PFU rMVAGn 1 dose in BALB/c mice *via* IP route 10^7^ PFU rMVAGc 1 dose in BALB/c mice *via* IP route 10^7^ PFU rMVAGnGc 1 dose in BALB/c mice *via* IP route	Yes	ND	Yes	10^3^ PFU 56/74	BALB/c mice 60% BALB/c mice 80% BALB/c mice 100%	(López-Gil et al., 2020)
	Gn, Gc and Np from MP12	10^7^ PFU rMVAGnGc 2 dose at 2 week interval in BALB/c mice *via* IP route 10^7^ PFU rMVANp 2 dose at 2 week interval in BALB/c mice *via* IP route	NT Yes	Yes ND	Yes ND	10^3^ PFU 56/74 at day 19	BALB/c mice 100% BALB/c mice 0%	(López-Gil et al., 2013)
	GnGc from MP12	10^7^ PFU MVA-GnGc-NS1 2 doses at 3 weeks interval in BALB/c mice *via* IP route	NT	Yes	NT	500 PFU 56/74 at day 35	BALB/c mice 100%	(Calvo-Pinilla et al., 2020)
	Gn and Gc from ZH501	10^7^ PFU vCOGnGc 2 doses at week 0, 8 in CB6F1 mice *via* IM route 10^7^ PFU vCOGnGγ 2 doses at week 0, 8 in CB6F1 mice *via* IM route	NT	Yes	NT	10^3^ PFU ZH501 at week 22	CB6F1 mice mice 90% CB6F1 mice mice 60%	(Papin et al., 2011)
	Gn	10^5.9^ TCID_50_ rKS1-GN RVF 2 doses at day 0, 21 in goat *via* subcutaneously	NT	ND	Yes	NT	NT	(Ayari-Fakhfakh et al., 2018)
	Gn and Gc from ZH548M12	10^8^ PFU CAdVax-RVF 1 dose in CD-1 mice *via* IP route 10^8^ PFU CAdVax-RVF 2 doses at week 0, 10 in CD-1 mice *via* IP route	Yes	ND	ND	100 PFU ZH501 at week 11 100 PFU ZH501 at week 27	CD-1 mice 100%	(Holman et al., 2009)
	Gn and Gc from MP12	10^8^ IU ChAdOx1-GnGc 1 dose in BABL/c mice *via* IM route 10^8^ IU HAdV5- GnGc 1 dose in BABL/c mice *via* IM route	NT	Yes	Yes	1000 PFU 56/74 at day 58	BABL/c mice 100%	(Warimwe et al., 2013)
	Gn and Gc from MP12	10^9^ IU ChAdOx1-GnGc 1 dose in pregnant ewes *via* IM route 10^9^ IU ChAdOx1-GnGc 1 dose in goats *via* IM route	NT	Yes	NT	10^5^ TCID_50_ rec35/74 at day 21	Pregnant ewes NT Goats NT	(Stedman et al., 2019)
	Gn and Gc	2x10^7^ TCID_50_ NDV-GnGc 1 dose in lambs *via* IM route	NT	ND	NT	10^5^ TCID_50_ rec35/74 at day 19	Lambs NT	(Kortekaas et al., 2012)
DNA	peGn from ZH548	100μg eGn + 20μg pGM-CSF 3 doses at day 0, 21, 42 in BALB/c	Yes	Yes	Yes	10^4^ PFU ZH501 at day 60	BALB/c mice 100%	(Chrun et al., 2019)
	Gn from ZH548	2μg Gn + 0.5mg gold beads 3 doses at week 0, 3, 6 in BALB/c mice 2μg Gn-C3d + 0.5mg gold beads 3 doses at week 0, 3, 6 in BALB/c mice	Yes	Yes	ND	10^3^ PFU of ZH501 at week 8	BALB/c mice 80% BALB/c mice 100%	(Bhardwaj et al., 2010)
	NsmGnGc, GnGc and Np	100μg pCMV-M1 2 doses at 2 weeks interval in IFNAR−/− mice *via* IM route 100μg pCMV-M4 2 doses at 2 weeks interval in IFNAR−/− mice *via* IM route 100μg pCMV-N 2 doses at 2 weeks interval in IFNAR−/− mice *via* IM route	ND ND Yes	NT Yes ND	NT	2x10^4^ PFU of MP12 at day 19	IFNAR^−/−^ mice 0% IFNAR^−/−^ mice 100% IFNAR^−/−^ mice 43%	(Lorenzo et al., 2010)
	Gn and Gc from 1974-VNIIVViM	50μg phRVF/Gn/7 + 50μg phRVF/Gn/5 1 dose in BALB/c mice *via* IM route	Yes	Yes	NT	NT	NT	(Selina et al., 2020)
	Gn and Gc from 1974-VNIIVViM	50μg pCI-neo/Gc/3 + 50μg pCI-neo/Gn/1 1 dose in BALB/c mice *via* IM route	Yes	Yes	NT	NT	NT	(Kuskov et al., 2021)

ND indicates not detected, NT indicates not tested, PFU indicates plaque forming unit, FFU indicates focus-forming units, IU indicates infectious units. IP indicates intraperitoneal, IM indicate intramuscular.

### 3.2 Attenuated vaccines

Live-attenuated vaccines are typically generated by the mutation or deletion of sequences, which limit the speed and scale of viral replication in the host. As such, while these types of vaccines stimulate more robust immune responses, disadvantages include safety concerns, such as reversion to virulence, and are typically not administered to immunocompromised patients.

The modified Smithburn vaccine was generated *via* serial passage of RVFV in a mouse brains and embryonated chicken eggs, and then passaged in BHK-21 cells to finally formulate the attenuated vaccine. The Smithburn animal vaccine has been sold in Eastern Africa and Saudi Arabia (Faburay et al., 2017), but alpacas have been found dead after vaccine administration (Anthony et al., 2021). Clone-13 virus was isolated from human with a 69% truncation in the non-structural protein NSs (Muller et al., 1995), the vaccine was proven safe in livestock such as sheep, goats and cattle (Sindato et al., 2021), but the occurrence of fetus malformations and stillbirths mean that the vaccine can induce serious side effects (Makoschey et al., 2016).

Through 12 serial passages of RVFV in the presence of 5-fluorouracil in MRC-5 cells, the attenuated MP-12 vaccine was obtained (Caplen et al., 1985), and was shown to protect 100% of mice from RVFV challenge after one dose (Morrill et al., 2022). MP-12 was found to be resistant to reversion to virulence (Ikegami et al., 2015), temperature changes (Nishiyama et al., 2016), and reassortment with other known RVFV isolates (Ly et al., 2017a). Although MP-12 is attenuated by multiple M- and L-segment mutations, NSs is found to be still functional (Billecocq et al., 2008). To further characterize mutations found in MP-12, 6 from 23 mutations of MP-12 were found *via* high-thoroughput sequencing to be derived from the parental RVFV ZH548 (Lokugamage et al., 2012). Continued passage of MP-12 on Vero cells showed that the re-amplification of rescued recombinant MP-12 (rMP-12) led to an increase in genetic subpopulations, affecting the viral phenotype *via* amino acid substitutions in the NSs gene, even though there was increased immunogenicity and the vaccine was shown to be safe (Ikegami, 2021).

From past studies, it is clear that the mutation of NSs is important for the attenuation of live RVFV vaccines. After replacement of the rMP-12 NSs with a modified NSs from the Clone-13 strain (C13), it was shown that rMP-12-C13type induced high nAbs and no viremia was detected (Lihoradova and Ikegami, 2012). The replacement of C13 NSs with the NSs of Punta Toro virus and sandfly fever sicilian virus showed that specific-NSs antibody was not induced but both candidates have better immune responses (**Table 2**) (Lihoradova et al., 2013). In contrast, the replacement of rMP-12 NSs with that from Toscana virus resulted in 100% protection but also enhanced the neuroinvasiveness of the vaccine candidate (**Table 2**) (Indran et al., 2013). Interestingly, through the modification of NSs, the rMP-C13type or rMP-12-mPKRN167 (a dominant-negative form of mouse dsRNA-dependent protein kinase in place of NSs) vaccine could protect 70% or 80% of mice at 20 min after a RVFV infection (**Table 2**) (Gowen et al., 2013). However, the truncated NSs protein seems to be related to the stimulation of a rapid protective innate immune response, suggesting that the functional inactivation of NSs plays an important role in the observed post-exposure efficacy (Fang et al., 2022). A study in which MP-12 was attenuated by the induction of 584 silent mutations within the N, NSs, M and L ORFs *via* reverse genetics showed that the protective efficacy remained 100% (**Table 2**) (Ly et al., 2017b).

Deletion vaccines based on the non-structural protein NSm were also explored as live-attenuated candidates, in which vaccinated livestock were protected from viremia and liver disease, with a significant decline in virus transmission among mosquitoes (**Table 2**) (Bird et al., 2008; Smith et al., 2018; Campbell et al., 2022; Ikegami et al., 2022). Serious side-effects, such as abortion or fetal malformation, were not observed (Weingartl et al., 2014).

Single-cycle replication-competent MP-12 (scMP-12), which lacks an endoplasmic reticulum retrieval signal and is defective for membrane fusion function, and RVFV-4s, which was generated by the splitting of the M segment open reading frame have shown better immunogenicity, protective efficacy and safety compared with MP-12 (**Table 2**) (Murakami et al., 2014; Wichgers Schreur et al., 2014b; Terasaki et al., 2018; Wichgers Schreur et al., 2021a; Wichgers Schreur et al., 2022). In addition, a favipiravir-mutagenized RVFV variant showed a strong attenuation, in which reverse genetics analysis showed that the mutations G924S and A1303T on segment were the main cause of attenuation (Borrego et al., 2022).

### 3.3 Subunit vaccines

The envelope glycoproteins Gn and Gc are a prime choice as target antigens for the development of RVFV vaccines (Sherman et al., 2009). Through expression of the Gn ectodomain (eGn) and the full-length Gc with the recombinant bacmid system, the antigens were formulated with Montanide ISA-25 VG adjuvant to generate a subunit vaccine. This candidate was shown to elicit strong nAbs after one dose and can completely protect sheep and cattle from RVFV challenge (**Table 2**) (Faburay et al., 2014; Faburay et al., 2016; Wilson et al., 2021). Although eGn expressed with the Drosophila system with Stimune adjuvant vaccine elicits lower levels of specific IgG compared to Montanide (Faburay et al., 2014), the mice were also completely protected (**Table 2**) (de Boer et al., 2010). Aside from GP-based vaccines, a recombinant RVFV NP with Alhydrogel adjuvant vaccine was evaluated in mice, and results showed that it elicited an early IFN-β as well as Th-2 responses, but cannot completely prevent RVFV infection (Jansen van Vuren et al., 2011).

Compared to free antigens, antigens immobilized on scaffolds, such as nanoparticles, utilize the Gn head domain bound to the lumazine synthase-based multimeric protein scaffold particles (MPSPs), were shown to improve immunogenicity and reduce mortality in BALB/c, as well as protecting lambs from viremia and clinical signs after immunization (**Table 2**) (Wichgers Schreur et al., 2021b). Through the insertion of the Gn head protein into Gram-positive enhancer matrix (GEM), which are non-living and non-genetically modified Gram-positive bacterial cells, a bacterium-like particle vaccine was constructed. Interestingly, while the results showed that IgG and nAbs were elicited after immunization, the immunogenicity was not dose-dependent. The induction of specific memory T-cells demonstrate the possibility for long-term protection (**Table 2**) (Zhang et al., 2022).

### 3.4 VLPs/RLPs vaccines

A study used the Drosophila insect protein expression system to generate VLPs of RVFV. VLPs composed of Gn and Gc, combined with the Stimune adjuvant, were shown to prevent RVFV infection in BALB/c mice (**Table 2**) (de Boer et al., 2010). In 2016, Li et al. (2016) used recombinant baculovirus to express RVFV NP and GP, which assembled into VLPs. They further explored the immunogenicity of these VLPs, and found that although T/B-cell responses were induced by VLPs, the responses were weaker than those stimulated with VLPs combined with Freund’s adjuvant (**Table 2**) (Li et al., 2020). Chimeric VLPs containing RVFV NP, Gn and Gc, meanwhile including gag from retroviruses were also tested against RVFV, in which the protective efficacy of chimeric VLPs is superior to VLPs with NP and VLPs without NP, which suggests gag enhances the uniformity and quantity of VLPs and may also serve as an adjuvant (**Table 2**) (Mandell et al., 2010).

Aside from traditional VLPs, protein fusion between eGn and the transmembrane domain and cytosolic tail (TMD/CT) of influenza hemagglutinin (HA) resulted in eGn-HA VLPs, which were found to elicit Gn-specific antibody (**Table 2**) (Mbewana et al., 2019), and provides a new prospect for VLPs development. Recombinant NDV expressing GnGc were used to infect stable cell lines with RVFV S and L genome segments to generate nonspreading RVFV (NSR), in which this strategy was shown to 100% protect mice from RVFV challenge (**Table 2**) (Wichgers Schreur et al., 2014a).

### 3.5 Viral vectored vaccines

Recombinant MVA vaccines expressing GnGc, Gn or Gc was developed and tested on mice. The results showed that these candidates did not elicit nAbs and passive transfer of immune serum to naïve mice did not result in protection. However, these vaccines induced strong T-cell responses, in which RVFV challenge resulted in protection efficacies of 100%, 60% and 80%, respectively, for rMVA-GnGc, rMVA-Gn and rMVA-Gc (**Table 2**) (López-Gil et al., 2020). In contrast, Lopez-Gil et al (López-Gil et al., 2013). demonstrated that the rMVA-NP vaccine did not induce IFN-γ and did not protect mice against a RVFV challenge (**Table 2**). The rMVA-GnGc(RVFV)-NS1(Bluetongue) demonstrated B-cell responses leading to survival against RVFV challenge in mice, but did not inhibit RVFV infection in vaccinated sheep (**Table 2**) (Calvo-Pinilla et al., 2020). Busquets et al. (2014) and Lorenzo et al. (2018) confirmed these results in sheep studies. From the above results, rMVA-GnGc is a promising potential candidate vaccine against RVFV.

Other poxvirus-vectored vaccines were also developed to investigate their efficacy against RVFV. A study used recombinant vaccinia viruses (rVACVs) to express RVFV GnGc or GnGc-IFNγ. Interestingly, the efficacy of the latter was lower, suggesting that the expression of IFN-γ may have played a role in reducing the replication of the recombinant pox-vectored vaccine (**Table 2**) (Papin et al., 2011). Based on KS1 recombination capripoxvirus (CPV) vector, a rKS1/RVFV-GnGc vaccine was developed, shown to induce high nAbs titers, and protected mice and sheep from RVFV challenge (Soi et al., 2010). To further understand the protective mechanism of the KS1/CPV-RVFV vaccine, Ayari-Fakhfakh et al. (2018) studied rKS1/CPV-Gn and found that the vaccine induced IL-4 and decreased IFN-γ in goats, suggesting the CD4^+^ T-cells are the main components of vaccine-induced rKS1/CPV-Gn immune response (**Table 2**).

The complex adenovirus (CAdVax) vector is a non-replicating system designed to avoid preexisting immune responses, which has hampered the efficacy of Ad5-vectored vaccines in the past. A CAdVax-GnGc vaccine was found to elicit strong GP-specific IgG antibody with 100% protection (**Table 2**) (Holman et al., 2009). Chimpanzee adenovirus vectors (ChAdOx) was another vector tested, and although ChAdOx1-GnGc was found to induce lower nAbs and T-cell response compared to Ad-5-GnGc, and both were found to have 100% protection from RVFV challenge (**Table 2**) (Warimwe et al., 2013). Warimwe et al (Stedman et al., 2019). further investigated ChAdOx1-GnGc efficacy and safety on sheep and goats, and showed that the candidates could inhibit RVFV infection in 100% of sheep and their fetuses, but not as protective in goat fetuses, suggesting differences in key mechanisms of protection against fetal RVFV infection between sheep and goats (**Table 2**).

Additionally, a RVFV pseudovirus based-VSV was constructed and successfully applied in neutralizing assay studies (Ma et al., 2019), suggesting the RVFV GP could inserted into VSV viral particles, and could be used for vaccine studies in the future. The Newcastle disease virus (NDV) vector and herpesvirus type 1 (EHV-1) vector expressing GnGc was shown to protect sheep from RVFV by nAbs (**Table 2**) (Kortekaas et al., 2012; Said et al., 2017).

### 3.6 DNA vaccines

Chrun et al. (2019) explored the effectiveness of a DNA vaccine expressing eGn (pscDEC-eGn) with GM-CSF adjuvant, in which the vaccine was shown to demonstrate complete protection from RVFV challenge, but the immunogenicity varied between different animal species, in which a decrese in the levels of specific IgG antibody led to a reduction in protection efficacy in mice (**Table 2**). Through comparison of the pTR600-eGn with pTR600-eGn with C3d adjuvant vaccine candidates, the latter was found to be better than the former on inducing specific IgG and nAbs. Although both vaccines did not elicit a detectable T-cell response, 80% and 100% protection was observed for pTR600-eGn and pTR600-eGn-C3d, respectively (**Table 2**) (Bhardwaj et al., 2010), suggesting that the B-cell response plays an important role on protection and that C3d could be a suitable adjuvant candidate.

Another study evaluated the protective efficacy of pCMV-NSmGnGc (0%), pCMV-GnGc (100%) and pCMV-NP (50%) against a RVFV challenge. It was found that a mixture of pCMV-NSmGnGc and pCMV-NP did not improve the protective efficacy of pCMV-NSmGnGc (**Table 2**) (Lorenzo et al., 2010), suggesting that the presence of NSm could impede the presentation process for the other antigens, and downregulate immune responses (Won et al., 2007).

Biodegradable alginate (ALG)/poly-L-lysine (PLL) microcapsules (MC), which are polycations used for fabrication of multilayer microcapsules, have been used to deliver DNA vaccines expressing Gn and Gc against RVFV, and results showed that this system enhanced specific-antibody and nAbs titers (**Table 2**) (Selina et al., 2020). The nanoparticles poly(N-vinylpyrrolidone) (PVP) can form complexes both with low molecular weight substances and polymers, and found to enhance humoral responses against Gn and Gc (**Table 2**) (Kuskov et al., 2021).

## 4 Vaccines against HTNV

In the 1950s, hemorrhagic fever with renal syndrome (HFRS) was reported. The causative pathogen, HTNV, was successfully isolated *via* serial passage in host rodents (*Apodemus agrarius coreae*) *(* Lee et al., 1978). HTNV sheds by host excreta and spreads to humans by aerosols (Jonsson et al., 2010). HTNV is mainly prevalent in China and Korea, which causes severe hemorrhagic fevers with a mortality rate ranging from 5% to 10% in humans (Cho et al., 2002). Four strains of HTNV, HTN76-118, AA57, KHF4 and KHF5, have been isolated from the host rodents and both caused hantavirus pulmonary syndrome-like disease in mice (Lee et al., 1978; Seto et al., 2012; Wei et al., 2022). Several vaccines against HTNV, based on the earliest 76-118 strain, have been developed and widely used, or are undergoing clinical evaluation.

### 4.1 Inactivated vaccines

Hantavax vaccine, the first vaccine developed against HTNV, was prepared from infected sucking mouse brains (Cho and Howard, 1999). Specific antibodies induced by Hantavax do not last for a long period and boosts are necessary to induce immune responses and maintain protection (Cho et al., 2002). Only 62% subjects showed seroconversion at 30 days after the first immunization and increased to 97% after a boost. The presence of nAbs was also boosted (from 13% to 75% of subjects) by the second immunization. However, only 50% of subjects were nAb-positive after 1 year (Cho et al., 2002). Current research on inactivated HTNV vaccines mainly focuses on the generation of robust long-term immune responses over a two-dose immunization strategy (**Table 3**) (Song et al., 2016; Song et al., 2020). Aside from Hantavax, two bivalent vaccines have also been developed against HTNV, one which also targets Seoul virus (SEOV) (Li et al., 2017) and the other also targeting Puumala virus (PUUV) (Cho et al., 2002). The HTNV-SEOV bivalent inactivated vaccine can induce high HTNV-NP specific IgG (over 1:1024) and last over 33 months after a three-dose immunization scheme.

**Table 3:** Summary of Hantaan vaccine development in animals or volunteers.

Vaccine type	Antigen origin	Dose	Specific antibody	Neutralizing antibody	T-cell response	Challenge	Efficacy survival	Reference
Inactivated	Hantavax^®^ from Green Cross Corporation	0.125 ml of virus 4 doses one month and 11-month intervals in volunteers ≥19 age	Yes	Yes	NT	NT	NT	(Song et al., 2020)
	Hantavax™ from Green Cross Corporation	0.125 ml of viru first dose at month 0, second dose at month 1, third dose at month 13 in volunteers aged 19-75 *via* IM or subcutaneous route	Yes	Yes	NT	NT	NT	(Song et al., 2016)
VLPs	N from Fojnica	Primary dose 20μg at day 0, second dose 10μg at day 10 and third dose 20μg at day 19 HBcdHTN120 with Freund’s adjuvant in four BALB/c mice and three C57BL/6 mice *via* IM and subcutaneous route	Yes	NT	NT	NT	NT	(Geldmacher et al., 2004; Geldmacher et al., 2005)
	Gn, Gc and Np from A9 strain	120μg HTN-VLPs 2 doses at day0, 14 in C57BL/6C mice *via* IM and subcutaneous route	Yes	Yes	Yes	NT	NT	(Li et al., 2010)
	M and S segment	100μg CD40L-VLP 1 dose in C57BL/6 mice *via* IP route 100μg Gn-CSF-VLP 1 dose in C57BL/6 mice *via* IP route 100μg HTNV-VLP 1 dose in C57BL/6 mice *via* IP route	Yes	Yes	Yes	Yes	C57BL/6 mice NT	(Ying et al., 2016)
Peptide	Gp peptide form HTNV 76-118	50 μg HLA-A*0201-restricted peptides with incomplete Freund’s adjuvant 3 doses at 10 days interval in HLA-A2.1/K^b^ Tg mice *via* subcutaneous route	NT	NT	Yes	10^5^ TCID_50_ HTNV 76-118 at day 40	HLA-A2.1/K^b^ Tg mice NT	(Tang et al., 2017)
	Gp peptide form HTNV 76-118 strain	50 μg PADRE-VV9 + 30 μg the N-terminal fragment N333 (aa22-aa355) of murine gp96 with complete Freund’s adjuvant in HLA-A2.1/K^b^ Tg mice *via* subcutaneous route 50μg VY9+gp96 with incomplete Freund’s adjuvant 3 doses in HLA-A2.1/K^b^ Tg mice *via* subcutaneous route	NT	NT	Yes	10^5^ TCID_50_ HTNV 76-118	HLA-A2.1/K^b^ Tg mice NT	(Ma et al., 2020)

ND indicates not detected, NT indicates not tested, IP indicates intraperitoneal, IM indicate intramuscular.

### 4.2 VLPs vaccines

The Hepatitis B virus (HBV) core protein is a platform that has been used for several VLP-based vaccine development (Zuckerman et al., 2001; Geldmacher et al., 2004; Geldmacher et al., 2005), not only against HBV but also against other pathogens, by chemical or recombinant linkage to other antigens (Jegerlehner et al., 2002; Geldmacher et al., 2004; Geldmacher et al., 2005). In one study, Geldmacher et al. (2004) inserted the 120 amino-terminal aa of HTNV N protein (NP) into a carboxyl-terminal truncated HBV core protein. This recombinant protein can form a chimeric VLP on which the epitope of HTNV is partially exposed. Mice were immunized with 20μg VLP as a prime, followed with 10μg and 20μg boost at days 10 and 19, respectively. Both BALB/c and C57BL/6 mice had robust levels of specific antibodies against HTNV NP at day 28. The titer of HTNV NP-specific antibody is approximately 1:10^5^ and 1:10^4^ in BALB/c and C57BL/6 mice, respectively. Additionally, the induced antibody also had a strong cross-reaction to Dobrava virus (DOBV) and PUUV (**Table 3**). All IgG subclasses were induced, in which IgG1 is almost two orders of magnitude higher than IgG2a, indicating a Th2-biased response (**Table 3**) (Finkelman et al., 1990; Geldmacher et al., 2005).

However, the pre-existing immunity in humans against the HBV core protein may interfere the function of an HBV-based vaccine. Recent investigations on HTNV VLP-based vaccines are mainly focused on complete HTN-VLP and its modification, which consists of NP and GP (Gn and Gc) (Finkelman et al., 1990; Li et al., 2010; Ying et al., 2016; Dong et al., 2019). In one study, Li et al. (2010) generated HTN-VLP by co-expressing HTNV NP, Gn and Gc in Chinese hamster ovary (CHO) cells (**Table 3**). C57BL/6 mice were immunized with two doses of 120μg HTN-VLP with a two-week interval. The anti-NP IgG was boosted after the second immunization, while anti-GP protein antibody was not. IgG levels were similar with those generated with the inactivated bivalent vaccine (YOUERJIAN^®^) (Li et al., 2010), while nAbs were both similar (NT50 from 1:16 to 1:32). However, HTN-VLP vaccines induced significantly stronger Th1-type immune responses at day 28 compared to inactivated HTNV vaccines (Li et al., 2010). The HTN-VLP was modified in the later work (Ying et al., 2016; Dong et al., 2019), in which Ying et al. (2016) incorporated the HTNV GP with CD40 ligand (CD40L) and granulocyte macrophage colony-stimulating factor (GM-CSF), which can enhance antigen presentation as well as lymphocyte activation and maturation. The two vectors expressing HTNV NP and modified GP were co-transfected to Dhfr cells and HTN-VLPs was purified. The mice were immunized with three doses of 100μg recombinant HTN-VLP in a 2-week interval. The NP/GP specific and nAbs induced by CD40L-VLP and GM-CSF-VLP were higher than the modified VLP and inactive vaccine. Moreover, the recombinant HTN-VLP group has a higher ratio of IgG2a/IgG1, indicating that the modification of HTN-VLP can change the immunoglobulin response into specific subtypes and trend to a Th1-type immune response. ELISpot assay results also provided evidence that both CD40L-VLP and GM-CSF-VLP can significantly increase the amount of splenocytes secreting IL-2 and IFN-γ, but not IL-4 and IL-10 (**Table 3**). The immune responses induced by the recombinant HTN-VLP were shown to protect mice against HTNV challenge and significantly decreased HTNV antigen levels in all organs. The CD40L-VLP animals are still able to maintain a nAbs titer of 1:80 at 6 months after the last immunization, which was almost two times higher than other vaccines (Dong et al., 2019).

### 4.3 Peptide vaccines

Peptide antigens are internalized and proteolyzed by antigen presenting cells and presented on the major histocompatibility complex (MHC) of the cell surface as the epitope to be recognized by T-cell receptors (Malonis et al., 2020). These epitopes are short peptides which can stimulate T-cells and activate cellular immune responses. Based on this, several peptide vaccines against plasmodium, Hepatitis C Virus (HCV), influenza virus and HIV-1 have been developed (Malonis et al., 2020).

Tang et al. (2017) predicted seven HTNV GP 9mer epitopes by Bioinformatics and Molecular Analysis Section and SYFPEITHI database, and synthesized the HTNV peptide/HLA-A^*^0201 tetramers as HTNV peptide vaccine candidates (**Table 3**). These epitopes were shown to be recognized by CD8^+^ T-cell from HFRS patients. Each epitope was prepared as a separate vaccine for mice immunization. The peptide vaccines were found to protect mice against HTNV challenge by inducing a protective cytotoxic T-cell (CTL) response, which significantly decreased the viral RNA load in the liver, spleen, and kidneys. However, the effect of peptide vaccines was not superior to the inactivated HTNV vaccine. Therefore, the authors modified the peptide vaccine and synthesized a linear multi-epitope peptide instead (**Table 3**) (Ma et al., 2020). The new peptide vaccine linked the HTNV CTL epitope (VV9) with pan HLA-DR-binding epitope and significantly increased the number of HTNV-specific IFN-γ secreting CD8^+^ T-cells. The protective effect of the vaccine was further improved by the modification, which reduced the viral RNA load in spleen. However, the activation of CD4^+^ T-cells and long-term protection were not tested with these candidates.

## 5 Prospects and perspectives

Despite the discovery of CCHFV, RVFV and HTNV over half a century ago, there remains a dearth of approved specific vaccines for human and livestock use. Aside from limited access to high biosafety level laboratories, which is required for experimental manipulation with live versions of these viruses, other factors complicating the rapid development of specific prophylaxis for these pathogens include: 1) the genetic diversity of these viruses due to the high mutation rates associated with RNA viruses and the segmented nature of bunyaviruses allowing for recombination, 2) the lack of a robust, immunocompetent animal model accurately recapitulating the hallmark disease symptoms observed in humans, and 3) the safety and efficacy of vaccines need to be better characterized.

An ideal vaccine is one that could protect humans from several (or all) genotypes of a specific pathogen. For instance, the seven CCHFV genotypes differ as much as 20%, 31% and 22% in the S, M and L segments, respectively, at the nucleotide sequence level (Bente et al., 2013). The increased geographical distribution of ticks carrying CCHFV, due to global warming, potentially facilitates the spread of different CCHFV subtypes to other regions (Kariwa et al., 2012; Fillâtre et al., 2019; Garrison et al., 2019). As such, a candidate vaccine effective against multiple genotypes of CCHFV would be welcomed. However, few studies have been completed thus far on the cross-protection of CCHFV vaccines, so in order to better prevent future outbreaks of CCHFV, investigating cross-reactive and potentially protective immune responses between CCHFV subtypes should be a topic of focus in the future. From a diagnostics viewpoint, it has been shown that the NP of CCHFV has the potential to detect different genotypes of CCHFV due to a more conserved sequence, which potentially supports cross-protection (Rangunwala et al., 2014; Shrivastava et al., 2021). Leventhal et al. showed that CCHFV NP from Hoti strain could protect 100% of mice from a challenge with the UG3010 strain (Leventhal et al., 2022).

In contrast, for 33 different strains of RVFV, NP shown 3.5% variable and 2.8% changes in amino acid of Gn and Gc (Ikegami, 2012), multiple studies have investigated the cross protection of RVFV vaccines, but the results were suboptimal (**Table 2**) and further vaccine optimization and investigations are needed. Considering that HTNV is more conserved and that the amino acid similarity of S, M and L segments between the A9 strain and 76-118 strain are 96.7%, 95.4% and 95.6%, respectively, also specify the biggest divergence of HTNV (Kariwa et al., 2012), it may be possible to develop a cross-protective vaccine against HTNV. Regardless, the ability of candidate vaccines to stimulate cross-protective immune responses against multiple pathogen genotypes should be a field of investigation in the future.

Regarding animal models, CCHFV does not cause disease and death in most immunocompetent, small adult animals such as mice, rats and hamster, so type I IFN signal pathway-related knockout mice have been used instead. Disease leading to partial to full lethality have been observed after a CCHFV challenge (Garrison et al., 2019), but the drawback of such animal models is the lack of ability to generate a complete immune response, which does not allow the effect of the vaccination to be fully characterized. For instance, CCHFV is sensitive to type I IFN (Rodriguez et al., 2022), but type I IFN effects induced by the vaccine cannot be evaluated in this model. To address this weakness, a mouse-adapted variant of CCHFV (MA-CCHFV) was developed by serial passaging, in which challenge with MA-CCHFV resulted in symptoms similar to human CCHF such as hepatitis, weight loss and lethargy (Hawman et al., 2021b). The non-human primate (NHP) cynomolgus macaque model was also developed by infection of animals with the Kosova Hoti strain, and shown that similar hallmark signs of human Crimean-Cango hemorrhagic fever disease, such as shock syndrome and coagulopathy (Haddock et al., 2018). So far, however, no clinical trials have approved after developing these two animal model.

In contrast, animal models for RVFV and HTNV are more well-characterized, because immunocompetent animals are susceptible to infection with these viruses. For example, RVFV infection in BALB/c mice could result in acute hepatic necrosis and meningoencephalitis and 100% death *via* intraperitoneal, subcutaneous, and aerosol challenge (Ross et al., 2012). In marmosets, RVFV infection could cause viremia, weight loss, kidney disease and 25-100% mortality (Smith et al., 2012). Through studies in these animal models, some vaccines have advanced to clinical trials, such as the ChAdOx1-based RVFV vaccine (Stedman et al., 2019). HTNV is known to be lethal for wild-type mice (in which the LD_50_ for C57BL/6 mice is approximately 60 PFU), in which infection causes encephalitis, pneumonia, and hepatitis (Wichmann et al., 2002; Wei et al., 2022). An activated HTNV vaccine (Hantavax) was eventually advanced to and completed Phase III clinical trials (Song et al., 2020). Therefore, the development of immunocompetent animal models is crucial for advancing vaccine candidates beyond the laboratory.

In order for vaccines to be approved for human use, safety and efficacy is a key consideration. Whether the vaccine causes side-effects, the rate of seroconversion, the strength of immunity over time needs to be evaluated in the long-term, with supporting data from a large number of experimental and clinical studies. This is especially important when using new emerging technologies such as the mRNA vaccine platform and silico-based antigen design. Billions of people were immunized with the mRNA vaccine during the SARS-CoV-2 pandemic, in which the data shows that mRNA vaccines appear to be safe and reliable (Fang et al., 2022). However, this vaccine platform will need to be characterized over time to generate data on long-term safety, immunogenicity and efficacy, especially when multiple boosters are required to maintain protection. Currently, vaccine candidates against the bunyaviruses discussed in this manuscript have not been evaluated for these parameters. In the future, it will be important to consider the points made above in order to produce optimal, well-characterized vaccines suitable for human use against these highly virulent bunyaviruses threatening human and animal health.

## Author contributions

TC: writing (first draft)-introduction, CCHFV, RVFV, discussion sections, and make tables. ZD: writing (first draft) HTNV and discussion sections. JL: writing-review outline, supervision, and reference support. GW: conceptualization, supervision, funding acquisition, and writing-review and editing. All authors contributed to the article and approved the submitted version.
